# Re-Cloning the N27 Dopamine Cell Line to Improve a Cell Culture Model of Parkinson's Disease

**DOI:** 10.1371/journal.pone.0160847

**Published:** 2016-08-11

**Authors:** Lu Gao, Wenbo Zhou, Breanna Symmes, Curt R. Freed

**Affiliations:** 1 Department of Human Anatomy and Histoembryology, School of Basic Medical Sciences, Fudan University, Shanghai, China; 2 Division of Clinical Pharmacology and Toxicology, Departments of Medicine and Pharmacology, the Neuroscience Program, the Gates Center for Stem Cell Research and Regenerative Medicine, and the Human Medical Genetics and Genomics Program, University of Colorado School of Medicine, Aurora, Colorado, United States of America; National Institutes of Health, UNITED STATES

## Abstract

Parkinson’s disease is characterized by the death of dopaminergic neurons in the substantia nigra. To understand the molecular mechanisms of the disease, an *in vitro* model is important. In the 1990s, we used the SV40 large T antigen to immortalize dopaminergic neurons derived from Embryonic Day 14 rat mesencephalon. We selected a clone for its high expression of dopaminergic neuron markers such as tyrosine hydroxylase (TH), and we named it 1RB3AN27 (N27). Because the original N27 cell line has been passaged many times, the line has become a mixture of cell types with highly variable expression of TH. In the current study, we have performed multiple rounds of clonal cultures and have identified a dopaminergic cell clone expressing high levels of TH and the dopamine transporter (DAT). We have named this new clone N27-A. Nearly 100% of N27-A cells express TH, DAT and Tuj1. Western blots have confirmed that N27-A cells have three to four times the levels of TH and DAT compared to the previous mixed population in N27. Further analysis has shown that the new clone expresses the dopamine neuron transcription factors Nurr1, En1, FoxA2 and Pitx3. The N27-A cells express the vesicular monoamine transporter (VMAT2), but do not express dopamine-beta-hydroxylase (DβH), the enzyme responsible for converting dopamine to norepinephrine. Functional analysis has shown that N27-A cells are more sensitive than N27 cells to neurotoxins taken up by the dopamine transporter such as 6-hydroxydopamine and 1-methyl-4-phenylpyridine (MPP+). The DAT inhibitor nomifensine can block MPP+ induced toxicity. The non-selective toxic effects of hydrogen peroxide were similar in both cell lines. The N27-A cells show dopamine release under basal and depolarization conditions. We conclude that the new N27-A clone of the immortalized rat dopaminergic cell line N27 should provide an improved *in vitro* model for Parkinson’s disease research.

## Introduction

Parkinson’s disease (PD) is the second most common neurodegenerative disease in the United States after Alzheimer’s [1–3]. PD is caused by the death of dopaminergic neurons in the substantia nigra pars compacta. Multiple factors contribute to neuron death including oxidative stress, abnormal protein aggregation, and loss of neuroprotective gene function [4–7]. To understand the molecular mechanisms of the disease, an *in vitro* model is important. Cultures of primary dopaminergic neurons derived from embryonic rat and mouse midbrain have been used frequently. Because primary cultures contain many cell types with fewer than 5% dopaminergic neurons, biochemical studies using these mixed cultures may produce misleading interpretations about dopamine neurons. Immortalized neurons offer an alternative. Other groups have developed mouse midbrain-derived MN9D cells [8–10], rat adrenal medulla-derived PC12 cells [11–15], human neuroblastoma cells SH-SY5Y [16–19], and BE(2)-M17 neuroblastoma cells [20, 21]. Each of these cell lines has dopaminergic properties which can sometimes be enhanced with chemical differentiation strategies.

In the 1990’s, we created a dopaminergic cell line from embryonic rat mesencephalic dopamine neurons immortalized with the SV40 large T antigen [22]. We named this clonal cell line 1RB3AN27 (N27). Biochemical analysis of the original N27 clone showed moderate concentrations of tyrosine hydroxylase (TH) and low levels of dopamine transporter (DAT). We found that the cells were sensitive to the neurotoxin 6-hydroxydopamine as well as to oxidative stress produced by hydrogen peroxide (H_2_O_2_). Over the years, we have distributed N27 cells to many labs around the world. N27 cells have been widely used with more than 100 papers using the N27 cell line for their dopaminergic properties as an *in vitro* model of PD, and for studying neurotoxicity, oxidative stress, neurodegeneration, and other molecular pathways [23–32].

Because the original N27 cell line from the 1990’s has been passaged many times, the line has mutated to become a mixture of cell types expressing highly variable levels of TH. The objective of this study was to isolate new N27 cell clones from the current mixed population. Clones were selected for high level expression of TH and DAT. Starting with a frozen vial of N27 cells which contained fewer than 5% TH+ cells, we performed clonal selection from single cells. After three rounds of clonal selection, we were able to isolate an N27 clone which has uniform high expression of TH and DAT. This N27-A clonal cell line has a morphologic phenotype that is much more neuronal than the starting mixed population.

## Materials and Methods

### N27 cell culture

N27 cells were cultured in RPMI 1640 medium with 10% fetal bovine serum, 2 mM L-glutamine, and 100 U/ml penicillin and streptomycin, as described previously [33]. The cells were cultured on 10-cm dishes for passaging, and on 48-well plates for TH immunostaining.

### N27 cell clonal culture

N27 cells were plated at 200 cells per 10-cm dish in the culture medium described above. After 10 days’ growth, cell colonies began to form. Forty-eight of these colonies were selected using 20 μl pipet tips under a dissection microscope and were plated into individual wells of a 48-well plate for expansion. After 7 days’ growth, cells from each well were dissociated with 0.05% trypsin/EDTA and plated into two identical 96-well plates. After cells became confluent, one 96-well plate was used for TH immunostaining to identify clones expressing high TH. Matching clones in the identical 96-well plate were selected and re-cloned in 10 cm dishes to further refine the high-TH expression of the clones. The whole cloning cycle was repeated three times. Finally, a single clone was chosen which had uniformly high expression of TH. The purified N27 cell clone (N27-A) was expanded through 24-well and 6-well plates and finally with large-scale production in10 cm dishes.

### Immunohistochemistry

For immunostaining, N27 cells were cultured on gelatin-coated 8-well chamber slides (BD Bioscience). When cells grew to 50% confluence, cultures were fixed with 4% paraformaldehyde for 30 minutes then rinsed with PBS. Cells were incubated with blocking buffer (5% normal goat serum in PBS, 0.1% Triton X-100 and 0.01% sodium azide) for 30 min, followed by incubation in the following primary antibodies diluted in blocking buffer overnight at room temperature: rabbit anti-TH (1:2000, Pel-Freez); mouse anti-TH (1:200, Millipore); rabbit anti-DAT (1:200, Santa Cruz Biotechnology); mouse anti-Tuj1 (1:200, Millipore); rabbit anti-Nurr1 (1:200, Santa Cruz Biotechnology); goat anti-En1 (1:200, Santa Cruz Biotechnology); mouse anti-FoxA2 (1:200, Santa Cruz Biotechnology); rabbit anti-Pitx3 (1:200, Millipore); rabbit anti-vesicular monoamine transporter 2 (VMAT2, 1:200, Sigma); rabbit anti-dopamine-beta-hydroxylase (DβH, 1:200, Millipore); rabbit anti- vesicular glutamate transporter 1 (vGluT1, 1:200, ThermoFisher); and rabbit anti-choline acetyltransferase (ChAT, 1:200, Millipore). Cells were rinsed with PBS followed by incubation with appropriate secondary antibodies conjugated with Alexa 488 or Alexa 544 (1:200 dilution in PBS, Invitrogen) in the dark for two hours at room temperature. Finally, cells were rinsed with PBS again and then examined for immunostaining under a fluorescence microscope.

### Western blotting

N27 and N27-A cells were collected in PBS followed by centrifugation to remove residual medium. Cell pellets were dissociated in a lysis buffer composed of 20 mM Tris-HCl, pH 7.5, 50 mM NaCl, 0.1% Triton X-100, and protease inhibitors. Protein concentrations were determined by the BCA method. One hundred μg of protein was separated on 12% Mini-Protean TGX gel (Bio-Rad) and transferred to a nitrocellulose membrane. Blots were probed with antibodies to TH (1:2000, Pel-Freez), DAT (1:1000, Santa Cruz Biotechnology) and β-actin (1:4000, Sigma). Blots were incubated with HRP-conjugated secondary antibody (1:10,000; Jackson Immuno Research), followed by chemiluminescent detection. The blot images were scanned and quantified by NIH ImageJ software. The TH and DAT band densities were normalized to β-actin bands.

### N27 cell growth rate

The purified N27-A and unpurified N27 cells were plated in 6-well plates at a density of 25,000 cells/well. On Days 1, 3, 5 and 7, three wells of cells were dissociated by 0.05% trypsin/EDTA. Cell density was determined by counting viable cells after trypan blue staining.

### N27 and N27-A cells treated with 6-OHDA, H_2_O_2_, or MPP+

Cells were plated in 24-well plates at a density of 10,000 cells/well for trypan blue staining, or in 96-well plates at 5,000 cells/well for MTT assay. Two days after plating, cells were incubated with chemicals producing oxidative stress at various doses for 24 hr: 6-hydroxydopamine (6-OHDA, 0–150 μM, RBI) prepared in PBS with 1% ascorbic acid, H_2_O_2_ (0–200 μM, Sigma), or MPP+ (0–1000 μM, Sigma) with or without 10 μM nomifensine (Tocris).

### Cell viability by trypan blue staining and MTT assay

For trypan blue staining, cells were dissociated with 0.05% trypsin/EDTA. After centrifugation, cell pellets were re-suspended in 100 μl Hank’s buffer. Five μl cells were mixed with 5 μl trypan blue solution (Invitrogen). Cell viability was determined by counting the viable cells and dead cells by phase contrast microscopy. For the MTT assay, methylthiazoletetrazolium (MTT, Sigma) was added to culture medium (final concentration at 0.4 mg/mL) and incubated for two hours. Culture medium was removed and precipitates were dissolved in 0.04 M HCl in isopropanol. Cell viability was measured by a plate reader at OD_590_.

### Dopamine release and measurement by ELISA

N27 and N27-A cells were cultured in 12-well plates. The culture medium was supplemented with 1x B27 (Life Technologies) which contains antioxidant to prevent dopamine oxidation. The cells were treated with or without 10 μM nomifensine for 24 hr. To induce depolarization, cells were treated with high KCl (50 mM) for 15 min and then culture medium was collected. After the culture medium was harvested, it was immediately treated with 1 mM EDTA and 4 mM sodium metabisulfite to stabilize dopamine. Dopamine concentrations were measured by a dopamine ELISA kit (Catalog Number KA3838, Abnova), following the manufacturer’s recommended protocol.

### Statistics

All experiments were repeated at least three times. Data were analyzed using multivariate ANOVA and Fisher LSD post hoc tests. Significance was set at *p*<0.05. Values are shown as mean ± SEM.

## Results

### Purification of N27 cells by clonal cultures

**Fig 1:**
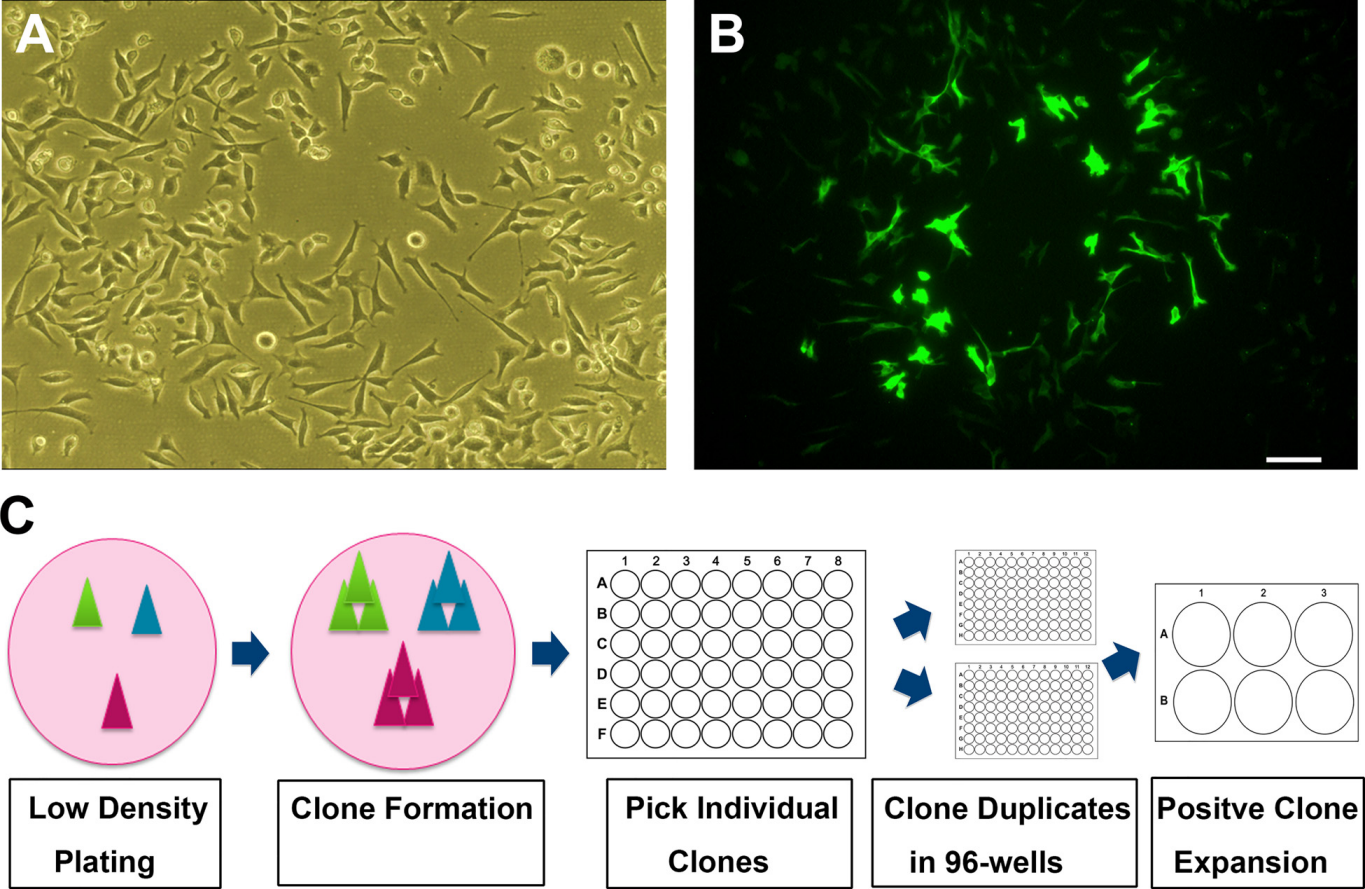
Process of N27 cells clonal purification. **(A):** Phase contrast image of unpurified N27 cells grown at low density. **(B):** Immunostaining for TH with green fluorescence in unpurified N27 cells. Shown is a cluster of cells exhibiting bright TH-positive staining. Most cells shown in phase contrast have no TH-immunoreactivity. **(C):** Schematic drawing of the clonal culture procedures for purifying N27-A cells. Cells were plated at low density to form individual colonies which were picked up and screened in 48- and 96-well plates. The positive clones were expanded in 6-well plates and 10-cm dishes. Bar, 20 μm for both **A** and **B**.

As shown in Fig 1A and 1B, the N27 cell line developed in the 1990’s has become a mixture of cell types (Fig 1A). Immnostaining for TH showed only small percentage of cells were TH positive. From a frozen vial of passage 22, we found a few clones of dividing cells that expressed high levels of TH (Fig 1B). These TH positive cells grew into a ring-shaped colony encircling the presumptive parent cell. Using a low-density plating strategy, we repeated colony selection to get a pure clone of cells with high expression of TH and DAT. We eventually found a clone which had nearly 100% TH-positive cells (Fig 1C). We have named this new clonal cell line N27-A.

### Growth properties of purified N27-A cells compared to unpurified N27 cells

**Fig 2:**
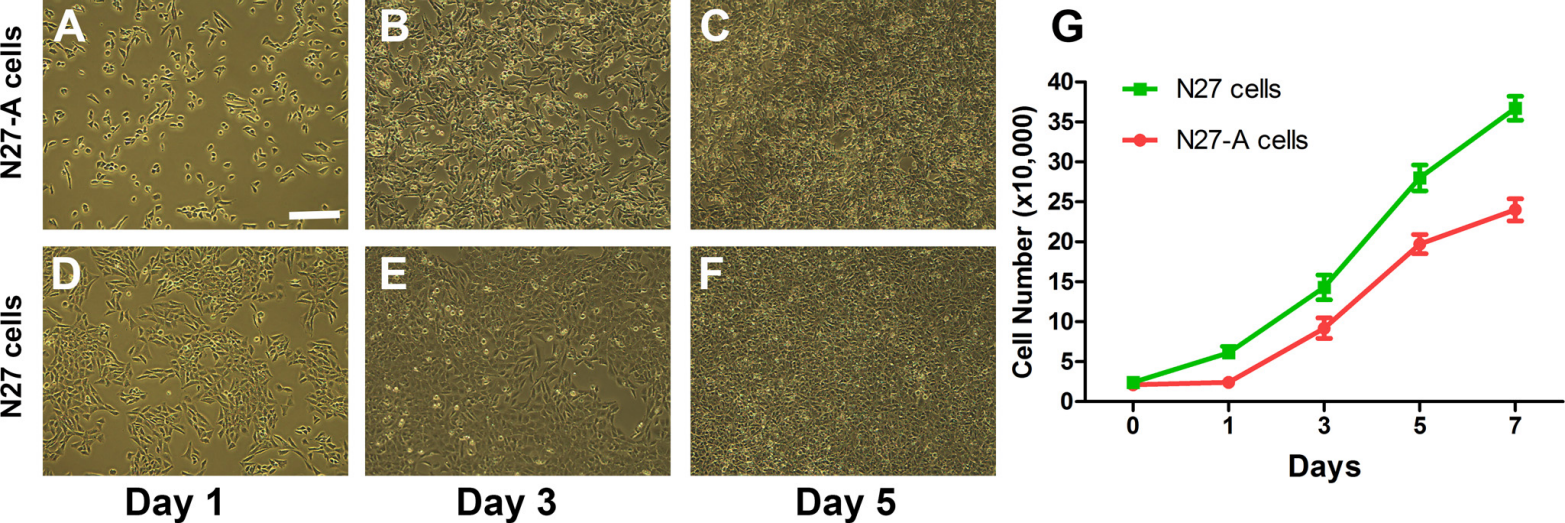
Growth properties of purified N27-A and unpurified N27 cells. **(A-F):** Both cell types were plated at 20,000 cells in each well of 6-well plates. Representative images were taken at Day 1, 3, and 5 for each cell type. Purified N27-A cells (**Images A-C**) grew more slowly than unpurified N27 cells (**Images D-F**). **(G)**: Growth charts of purified N27-A (red line) and unpurified N27 cells (green line) from Day 0 to Day 7. Data present the average cell number from two wells of purified and unpurified cells in three experiments (n = 6, each cell type). Bar, 20 μm for **A-F**.

We have compared the cell growth rate of purified N27-A cells and unpurified N27 cells. We plated both cell sources at 20,000 cells in each well of a 6-well plate on Day 0. Cultures were photographed at Days 1, 3, and 5. The purified N27-A cells grew more slowly than unpurified N27 cells, as shown in Fig 2A–2F. On Days 1, 3, 5, and 7, cells were dissociated and cell counts performed. As shown in Fig 2G, the doubling time for purified N27-A cells was about 36 hr compared to 24 hr for unpurified N27 cells, reflecting the rapid turnover of non-dopaminergic components of the mixed population of unpurified N27 cells.

### Purified N27 cells express phenotypic markers for dopamine neurons–TH and DAT

**Fig 3:**
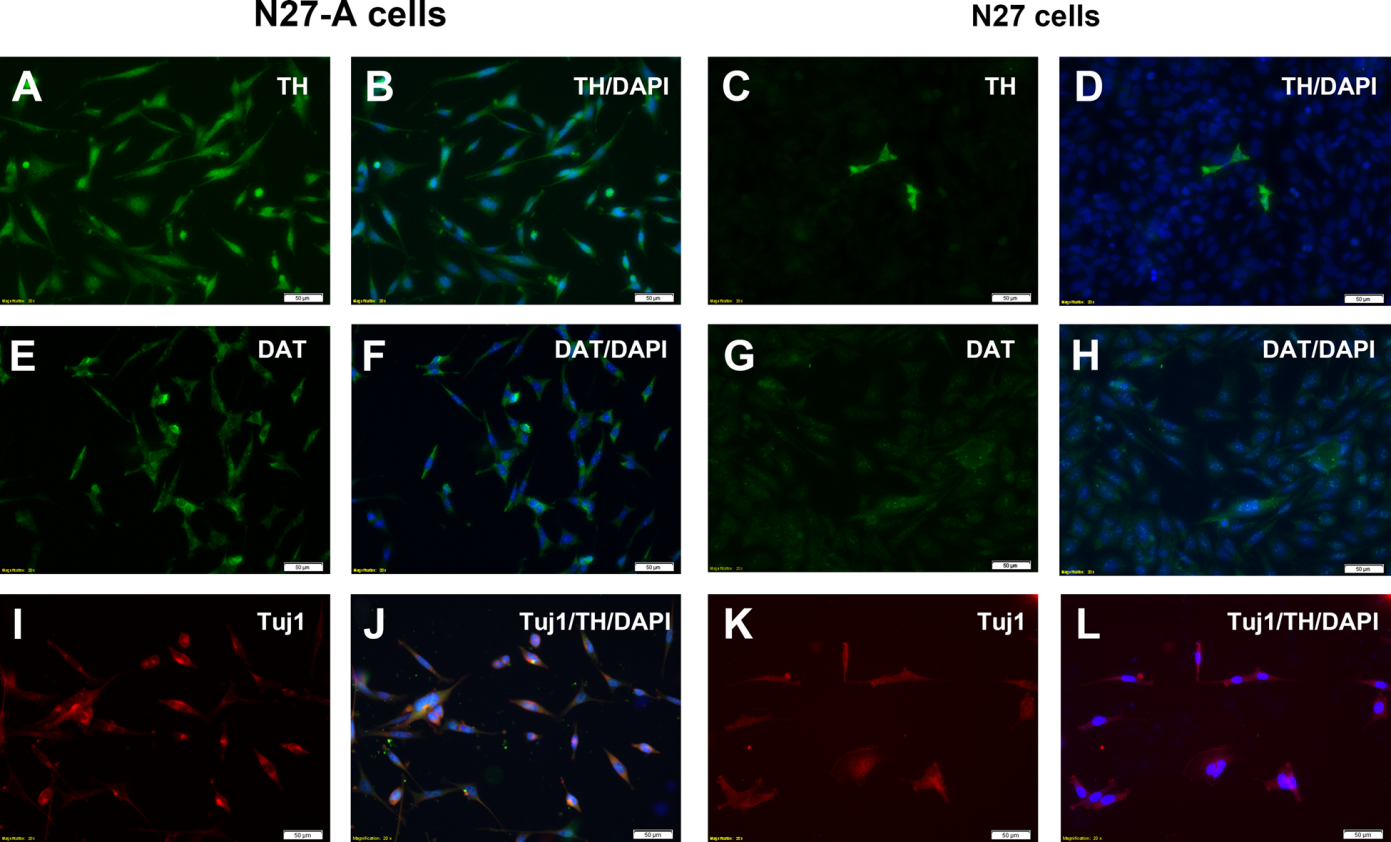
Immunocytochemistry of purified N27-A and unpurified N27 cells for dopamine neuron markers TH and DAT. The N27 cells were cultured on 8-well chamber slides and immunostained for the dopamine neuron markers TH **(A-D)** and DAT **(E-H).** Other wells were double-stained for TH and the neuronal marker Tuj1 **(I-L).** To image every cell in each well, the nuclear marker DAPI was added to all wells. **(A-B):** The purified N27-A clone showed strong TH staining in all cells as demonstrated by dual-staining with TH and DAPI. **(C-D):** The unpurified N27 cell mixture revealed that only a small fraction of the DAPI-labeled cells were TH positive. **(E-F):** In the purified N27-A clone, all cells had moderate DAT staining as shown with DAT and DAPI double staining. **(G-H):** In the unpurified N27 cell mixture, very few cells were positive for DAT immunostaining. **(I-J)**: In the purified N27-A clone, all cells were double-positive for Tuj1 and TH. **(K-L)**: While there were few TH-positive cells in the unpurified N27 cell mixture, most cells were Tuj1 positive, demonstrating that the mixed cell population was neuronal. Bar, 50 μm for **A-L**.

We characterized the newly purified N27-A cells by immunostaining for key dopamine neuron markers TH and DAT. Immunostaining for TH revealed that nearly 100% of purified N27-A cells were TH positive (Fig 3A and 3B), while unpurified N27 cells had only a small fractions of cells which were TH positive (Fig 3C and 3D). Immunostaining for DAT revealed that nearly all the purified N27-A cells were DAT positive (Fig 3E and 3F), while unpurified N27 cells only had a few cells positive for DAT (Fig 3G and 3H). To confirm that purified N27 cells were neuronal cells, we performed Tuj1 immunostaining. Results showed that both N27-A cells and the original N27 cells were all positive for Tuj1 (Fig 3I–3L), indicating that all N27 cells have neuronal characteristics.

### Purified N27-A cells express dopaminergic transcription factors Nurr1, En1, FoxA2 and Pitx3 as well as the vesicular monoamine transporter VMAT2

**Fig 4:**
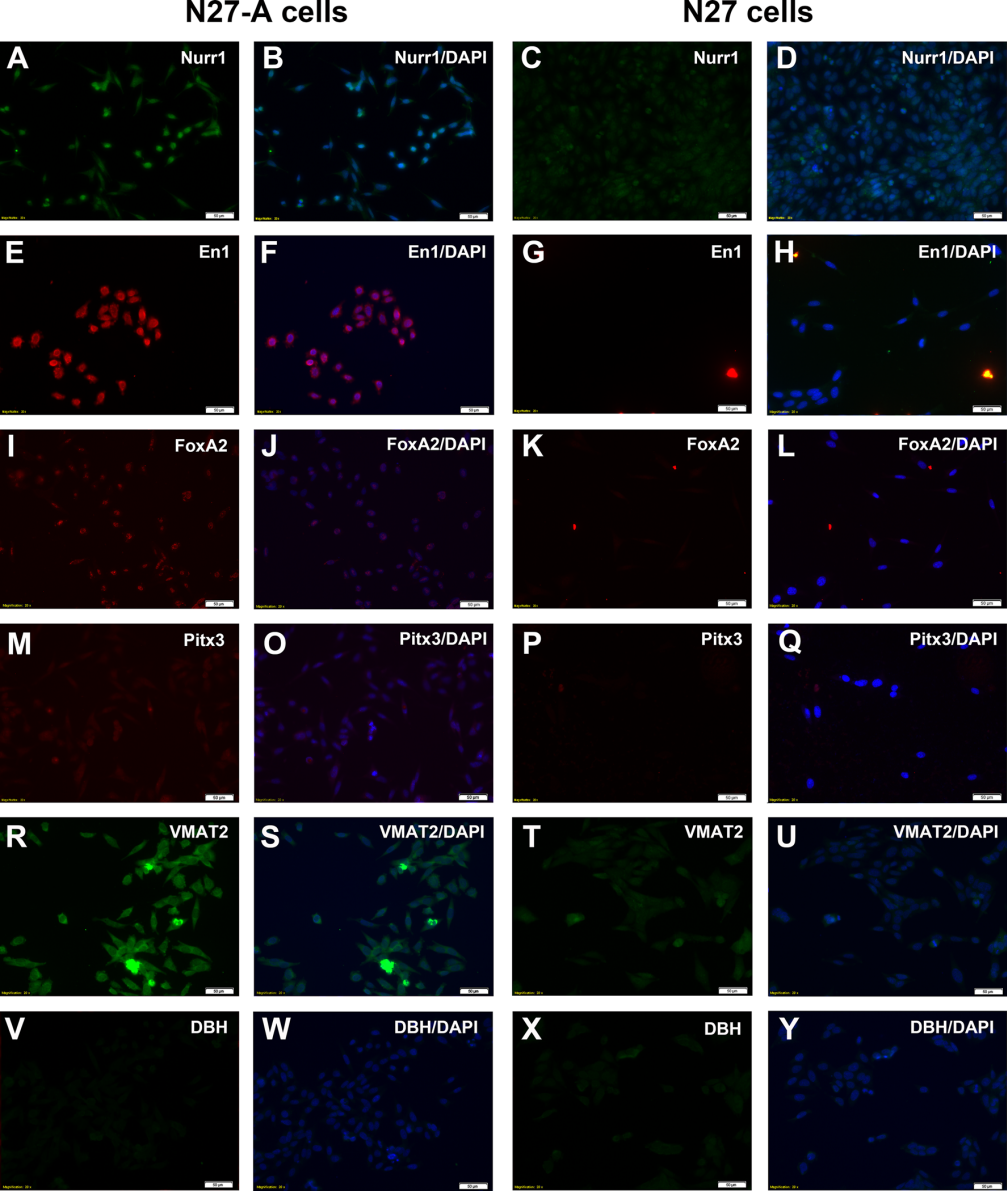
Dopamine neuron transcription factors (Nurr1, En1, FoxA2 and Pitx3) and VMAT2 and DβH in purified N27-A and unpurified N27 cell cultures. N27 cells were cultured on 8-well chamber slides and immunostained with the dopamine neuron transcription factors Nurr1 **(A-D)**, En1 **(E-H)**, FoxA2 **(I-L)** and Pitx3 **(M-Q),** as well as VMAT2 (**R-U**) and DβH (**V-Y**). Cells were also stained for the nuclear marker DAPI. **(A-B):** In N27-A clone, nearly all cells had strong Nurr1 staining as shown by Nurr1 and DAPI double staining. **(C-D)**: In unpurified N27 cells, only a small fraction of cells expressed Nurr1. **(E-F):** In N27-A cells, most cells had strong En1 staining. **(G-H)**: In unpurified N27 cells, nearly all cells were negative for En1 staining. **(I-J):** In N27-A clone, cells were moderately stained for FoxA2. **(K-L)**: In unpurified N27 cells, nearly all cells were FoxA2 negative. **(M-Q)**: In N27-A clone, cells were faintly stained for Pitx3. In unpurified N27 cells, no cells expressed Pitx3. (R-U): In N27-A clone, most cells were positive for VMAT2. In unpurified N27 cells, only a few cells were VMAT2-positive. (V-Y): In both N27-A and N27 clones, no cells were DβH-positive. Bar, 50 μm for **A-Y**.

To further demonstrate the dopaminergic character of purified N27-A cells, we immunostained them with transcription factors found in dopamine neurons: Nurr1, En1, FoxA2 and Pitx3. Immunostaining for Nurr1 showed that nearly all purified N27-A cells were positive for Nurr1 (Fig 4A and 4B). By contrast, there were no Nurr1-positive cells in the unpurified N27 cells (Fig 4C and 4D). Immunostaining for En1, FoxA2 and Pitx3 revealed some nuclei which were strongly positive for at least one of the factors, but many cells only had faint staining (Fig 4E–4O). Unpurified N27 cells had no nuclei positive for En1, FoxA2 and Pitx3 (Fig 4G–4Q). Furthermore, we have found that N27-A cells expressed the dopaminergic specific protein vesicular monoamine transporter VMAT2 (Fig 4R–4S), but not noradrenaline production enzyme dopamine-β-hydroxylase DβH which converts dopamine to noradrenaline (Fig 4V–4W). The original N27 cells had very weak expression of VMAT2 (Fig 4T–4U) and no staining of DβH (Fig 4X–4Y). To see if either cell line contains glutamaterigic or cholinergic cell markers, we have performed immunostaining with vGluT1 and ChAT antibodies in N27-A and N27 cells. Neither cell line had staining for vGluT1 or ChAT, indicating that N27 and N27-A cells do not have glutamatergic or cholinergic cell property.

### Western blots confirm that purified N27-A cells express higher levels of TH and DAT than unpurified N27 cells

**Fig 5:**
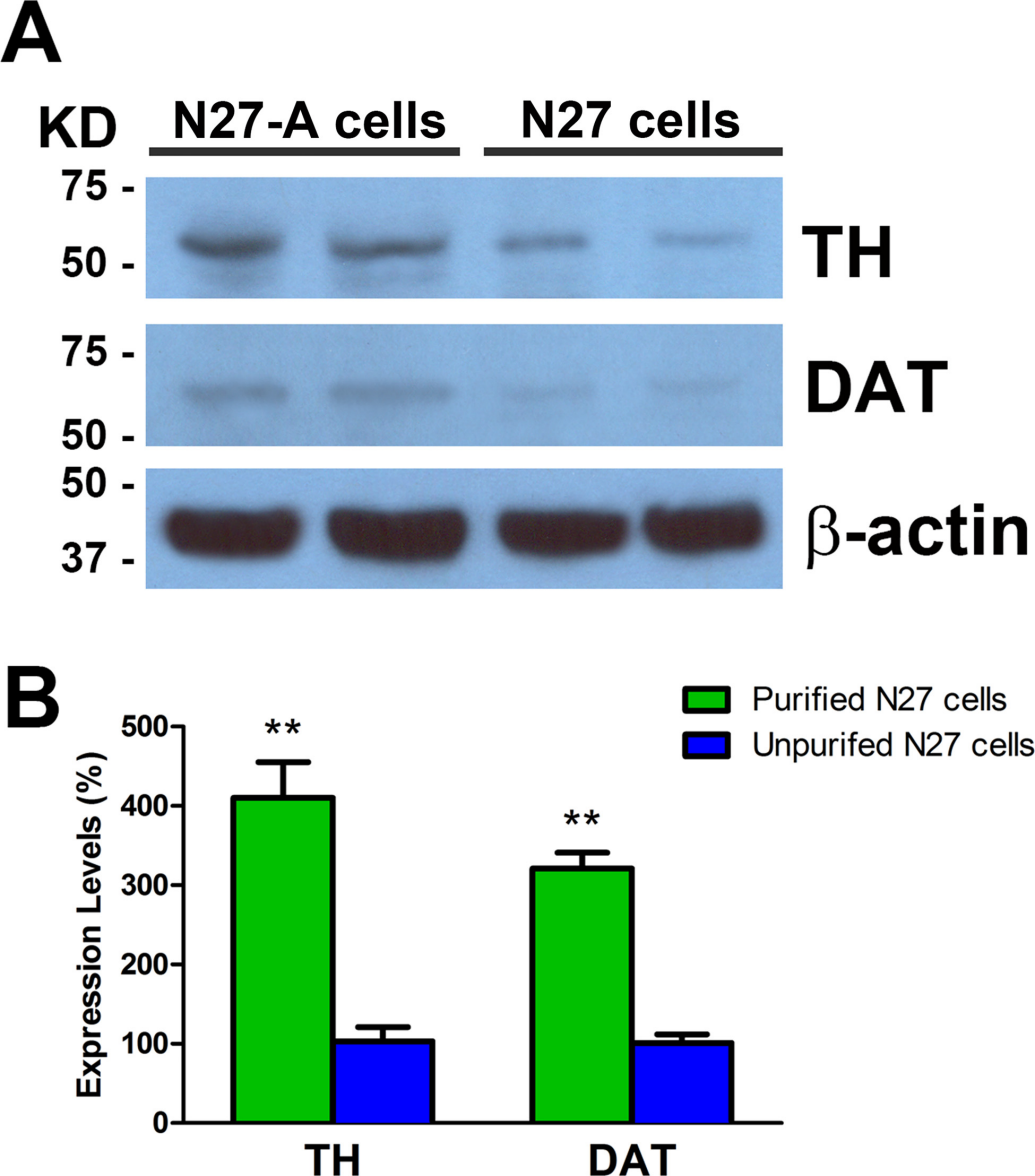
Western blots for TH and DAT in purified N27-A and unpurified N27 cells. **(A)**: Representative images show TH, DAT and β-actin Western blots from purified and unpurified N27 cells. Strong TH bands were seen in purified N27-A cells, while much reduced TH protein levels were seen in unpurified N27 cells. There were moderate DAT protein levels in purified N27-A cells but only faint DAT bands in unpurified N27 cells. **(B):** Quantification of TH and DAT Western blots relative to β-actin bands. The TH and DAT levels in unpurified N27 cells were set at 100%. Results show that purified N27-A cells have four-fold higher TH and three-fold higher DAT protein levels compared to unpurified N27 cells. (n = 6, **p<0.01)

Western blots were done to measure protein levels of TH and DAT in purified and unpurified N27 cells. Results showed that purified N27-A cells express approximately four-fold higher levels of TH compared to unpurified N27 cells (Fig 5A and 5B). Western blots for DAT revealed that purified neurons had three-fold higher DAT concentrations compared to unpurified N27 cells. The Western blot results provide quantitative confirmation of the immunohistochemical evidence for purified N27-A cells having high levels of TH and DAT.

### Purified N27-A cells are more sensitive to 6-OHDA than unpurified N27 cells

**Fig 6:**
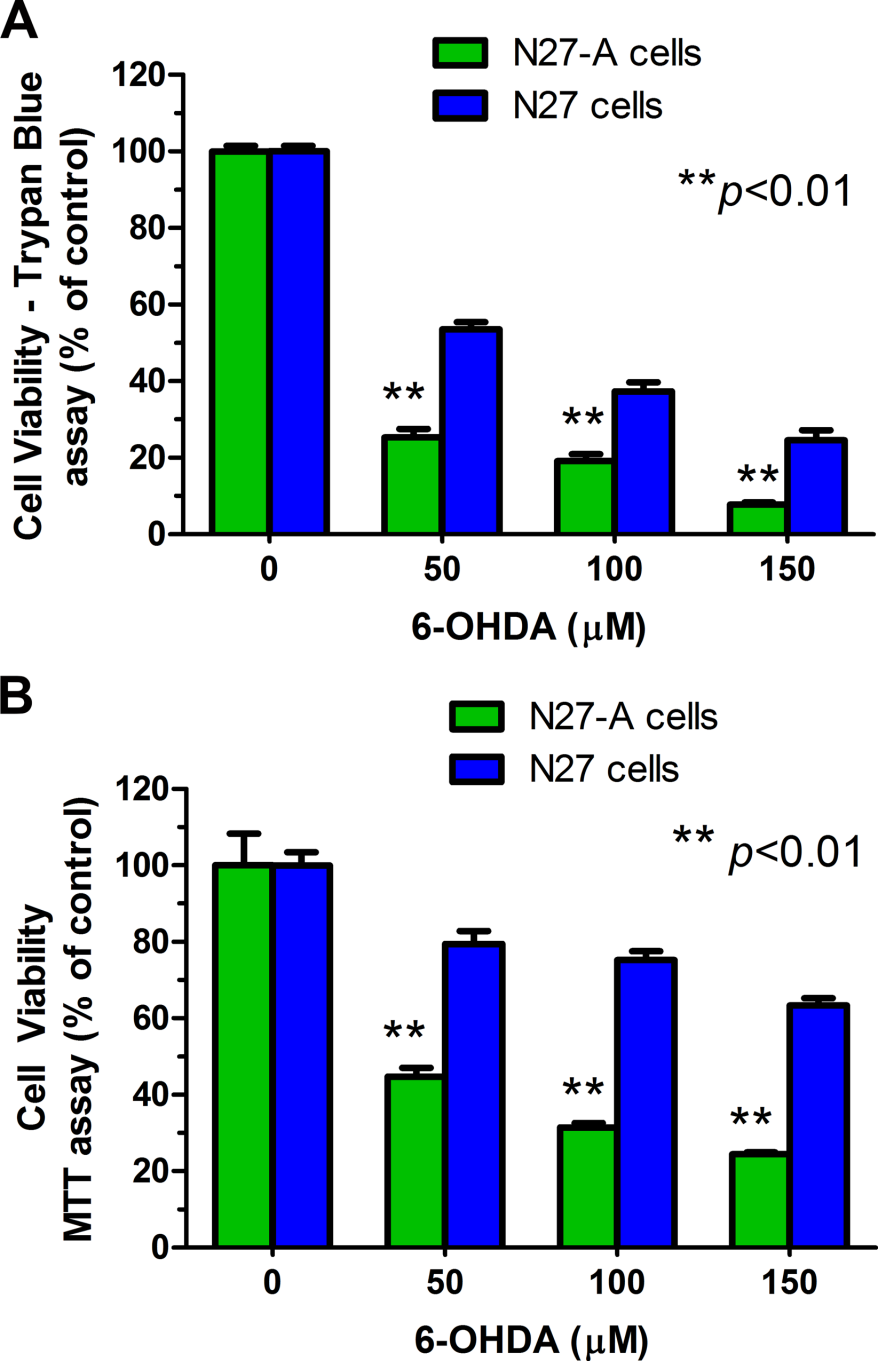
Purified N27-A cells are more sensitive to 6-OHDA toxicity than unpurified N27 cells. Purified N27-A and unpurified N27 cells were cultured in 24-well plates (for trypan blue staining) and 96-well plates (for MTT assay). Two days after plating, cells were treated with 0–150 μM of 6-OHDA for 24 hr. The cell viability was measured by trypan blue staining **(A)** and MTT assay **(B)**. **(A):** Cell viability data from trypan blue staining showed that there were significantly fewer viable cells in purified N27-A cultures compared to unpurified N27 cultures. **(B)**: Cell viability results from the MTT assay also showed that purified N27-A cells had greater cell death than unpurified N27 cells after exposure to 6-OHDA. Reduced cell survival in both assays indicate that purified N27-A cells are more sensitive to 6-OHDA than unpurified N27 cells (n = 12 for **A**, n = 15 for **B**, **p<0.01).

The neurotoxin 6-OHDA is selectively transported via the dopamine transporter (DAT). We compared toxicity of 6-OHDA in purified and unpurified N27 cells. All cultures were treated with 0–150 μM of 6-OHDA prepared in the stabilizing agent 1% ascorbic acid for 24 hr. Cell viability was determined by trypan blue staining (Fig 6A) and MTT assay (Fig 6B). We found that purified N27-A cells were significantly more sensitive to 6-OHDA-induced toxicity than unpurified cells (Fig 6A and 6B, **p<0.01). For example, exposure to 50 μM 6-OHDA led to 75% death of purified N27-A cells but only 50% death of unpurified cells (trypan blue exclusion). Similarly, the MTT assay showed more cell death in purified N27 neurons after 6-OHDA. These results indicate that the purified N27-A cells were more sensitive to a dopaminergic neurotoxin than unpurified N27 cells.

### Purified N27-A cells are more sensitive to MPP+ than unpurified N27 cells

**Fig 7:**
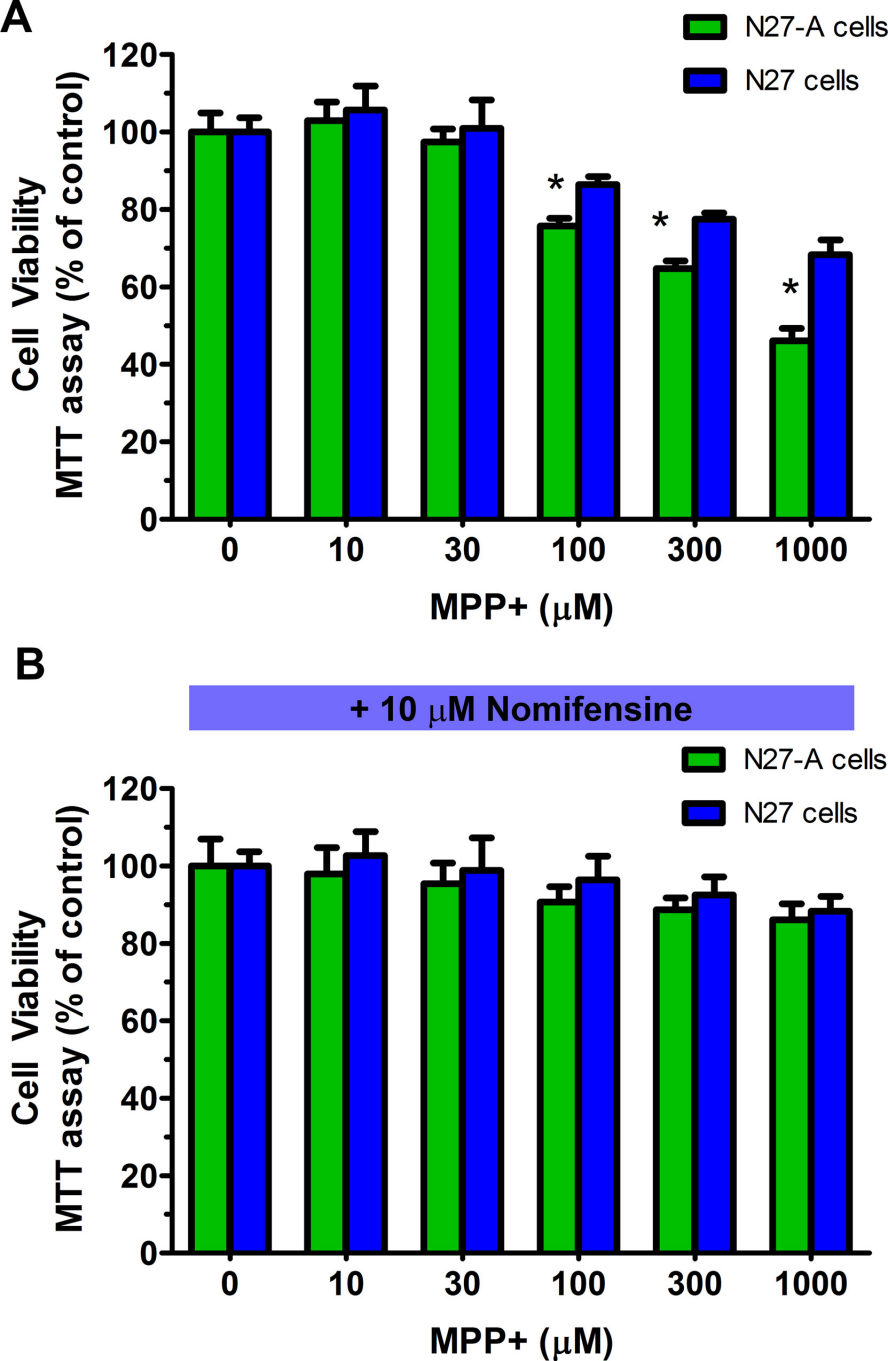
N27-A cells are more sensitive to MPP+ toxicity than unpurified N27 cells, and nomifensine can block MPP+ induced toxicity. Purified N27-A and unpurified N27 cells were cultured in 96-well plates at equal starting density. Two days after plating, cells were treated with 0–1000 μM of MPP+ for 24 hr. **(A):** Cell viability data from MTT assays showed that there was significantly greater cell death in N27-A cultures compared to that in N27 cultures after 100 μM to 1000 μM of MPP+ treatment (n = 12, *p<0.05). **(B)**: After nomifensine pre-treatment, exposure to MPP+ did not significantly reduce cell viability in either N27-A or N27 cells, even when MPP+ reached 1000 μM (n = 12, *p*>0.3).

The dopamine neurotoxin MPP+ is also selectively transported via the dopamine transporter DAT. We compared toxicity of MPP+ in purified and unpurified N27 cells. The cultures were treated with 0–1000 μM of MPP+ for 24 hr. Cell viability was determined by MTT assay (Fig 7A). We found that purified N27-A cells were significantly more sensitive to MPP+ induced toxicity than unpurified N27 cells (Fig 7A, *p<0.05), at 100 μM, 300 μM and 1000 μM of MPP+ treated cells. Furthermore, we have found that pre-treatment with 10 μM of the DAT inhibitor nomifensine can completely block the MPP+ toxicity (Fig 7B, *p*>0.3), even at 1000 μM of MPP+. These results indicate that the purified N27-A cells were more vulnerable to the dopaminergic neurotoxin MPP+ than unpurified N27 cells, and the toxicity is mediated by dopamine transporter.

### Purified and unpurified N27 cells are equally sensitive to H_2_O_2_ toxicity

**Fig 8:**
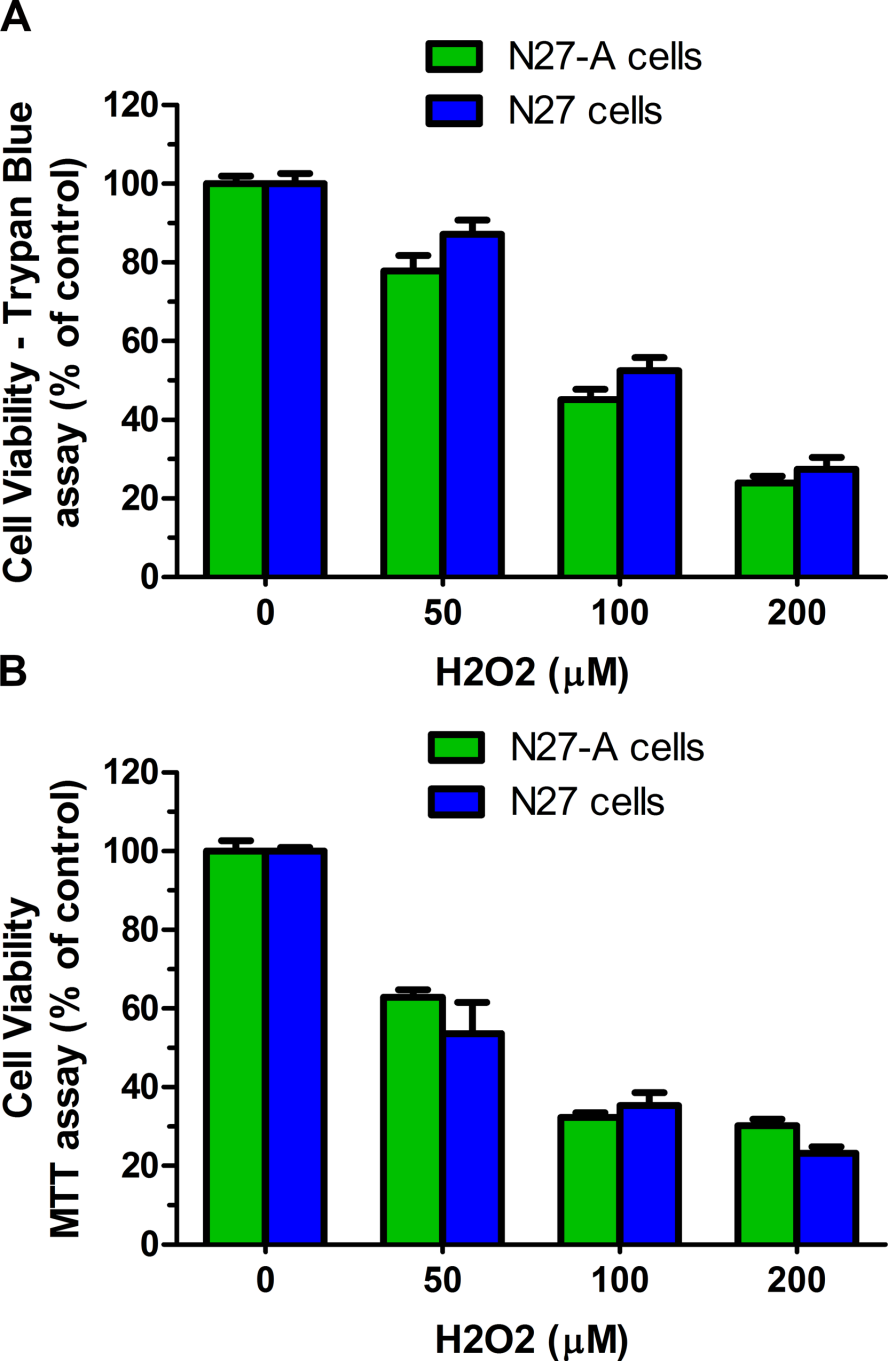
H_2_O_2_ toxicity is similar in purified and unpurified N27 cells. Purified N27-A cells and unpurified N27 cells were cultured in 24-well plates (for trypan blue staining) and 96-well plates (for MTT assay). Two days after plating, cells were treated with 0–200 μM of H_2_O_2_ for 24 hr. Cell viability was measured by trypan blue staining **(A)** and MTT assay **(B). (A):** Cell viability data from trypan blue staining showed similar numbers of viable cells in purified and unpurified N27 cells. **(B):** Cell viability results from the MTT assay revealed no significant differences in surviving cells from purified N27-A or unpurified N27 cells indicating that both cell sources had similar sensitivity to the non-specific toxicity of H_2_O_2_ (n = 12 for **A**, n = 15 for **B**, **p<0.01).

Hydrogen peroxide (H_2_O_2_) can produce lethal oxidative stress in most cells. We compared the toxicity profile of H_2_O_2_ in purified and unpurified N27 cells. N27 cells were treated with 0–200 μM of H_2_O_2_ for 24 hr. Cell viability was determined by trypan blue staining (Fig 8A) and MTT assay (Fig 8B). We found that H_2_O_2_ was equally toxic to purified N27-A cells and unpurified N27 cells (Fig 8A and 8B, *p*>0.2). Using the trypan blue viability assay, after 24 hr treatment with 100 μM of H_2_O_2_, both populations of cells had about 50% viable cells. Similarly, after 24 hr exposure to 50 μM of H_2_O_2_, the MTT assay revealed 50% viable cells in both purified and unpurified N27 cells. These results indicate that purified N27-A cells were equally sensitive to oxidative stress as were unpurified N27 cells.

### Purified N27-A can release dopamine and respond to depolarization

**Fig 9:**
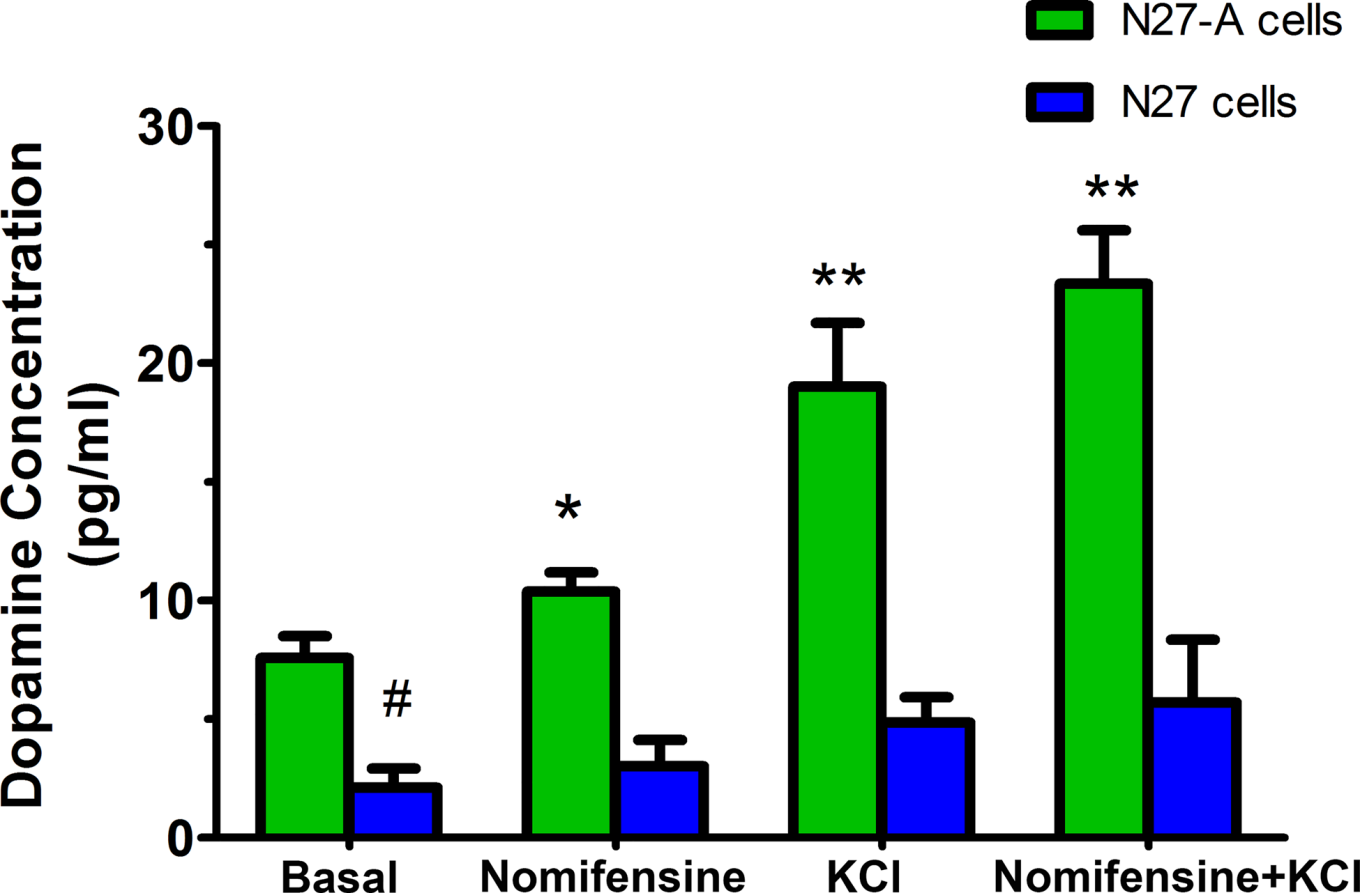
Dopamine release from N27-A and N27 cells. N27-A and N27 cells were cultured in 12-well plates and treated with or without 10 μM nomifensine for 24 hr. Under basal conditions, N27-A cells had about 3-fold of higher dopamine concentrations in the medium than N27 cells (n = 4, ^#^*p*<0.01). In N27-A cells, dopamine concentrations were significantly increased when cells were treated with nomifensine, potassium depolarization, or nomifensine plus potassium depolarization compared to basal conditions (n = 4, *p<0.05, **p<0.01). In unpurified N27 cells, there was only a slight increase in dopamine release under these three conditions (n = 4, *p*>0.1).

We have measured whether dopamine can be released from the purified N27-A cells and unpurified N27 cells. We have collected medium from basal conditions, and from cells treated with nomifensine, high KCl depolarization, and the combination of nomifensine and high KCl conditions. Results showed that dopamine was measurable in media under basal conditions in both N27-A and N27 cells. N27-A cells had 3-fold higher levels of dopamine than N27 cells (Fig 9, ^#^*p*<0.01). When cells were exposed to dopamine reuptake inhibition with nomifensine or cell depolarization using high potassium, dopamine release was much greater from N27-A cells compared to N27 cells. The combination of potassium plus nomifensine led to some additional release from N27-A cells (Fig 9, *p<0.05, **p<0.01). These data indicate the N27-A clonal cell line can synthesize and release dopamine. Transport of dopamine is via DAT as demonstrated by the effects of the DAT blocker, nomifensine.

## Discussion

In this study, we have used clonal culture methods on the established N27 cell line to derive a new clonal dopaminergic cell line which we have named N27-A. The N27 cell line has been used in more than 100 published experiments. Therefore, we decided that the research community would benefit from a new clonal form of the cell line. Because the purified N27-A cell clone was selected for homogenous expression of TH, nearly every cell expresses high levels of the TH protein. The N27-A cells also express DAT, a marker which is specific for dopamine neurons. N27-A cells also express the vesicular monoamine transporter VMAT2. Immunostaining results were confirmed by Western blots for TH and DAT. The new clone was tested for a number of transcription factors associated with dopamine neurons. These included Nurr1, En1, FoxA2, and Pitx3. Nearly all cells express high levels of Nurr1 which is an important regulator of TH expression [24, 34–36]. The transcription factors En1 [37, 38], FoxA2 [39, 40], and Pitx3 [41, 42] are also important for establishing and maintaining the dopamine neuron phenotype. N27-A cells express moderate levels of En1, FoxA2, and Pitx3, further validating their dopaminergic phenotype. Cells do not express dopamine-β-hydroxylase DβH, the enzyme that converts dopamine to noradrenaline. We have ruled out the presence of glutamatergic and cholinergic cells in the N27-A clone. We have demonstrated that N27-A cells can synthesize and release dopamine, and respond to depolarization.

To demonstrate the importance of the dopamine transporter (DAT) in the physiology of authentic dopamine neurons, we tested the dopamine neuron-specific toxin 6-OHDA and MPP+. Because purified N27-A cells express more DAT than unpurified cells, we expected greater uptake of 6-OHDA in the purified cells and therefore greater toxicity in those cells. We found that purified N27-A cells are more sensitive to cell death by 6-OHDA and MPP+ than unpurified N27 cells. Importantly, MPP+ induced toxicity can be blocked by nomifensine, indicating DAT is mediating the MPP+ toxicity. By contrast, the non-selective oxidative stress produced by H_2_O_2_ was equally toxic to both purified and unpurified cells.

The doubling time of purified N27-A cells was slower than for the original unpurified N27 cells, 36 hr versus 24 hr. It is likely that the mixed population of the unpurified N27 cells had subclones that were dividing more rapidly and were responsible for diluting out the strongly dopaminergic clones contained in the original N27 cultures and regained with the N27-A clone.

Our N27 rat dopaminergic cell line was originally derived in the 1990’s by immortalizing embryonic rat dopamine neurons with the SV40 large-T antigen. During many passages over 20 years, the original N27 cell line became a mixture of cell types with less than 5% TH-positive dopamine neurons. Our successful isolation of the new N27-A cell clone with expression of TH, DAT, VMAT2, dopaminergic transcription factors, dopaminergic storage protein, and functional dopamine synthesis and release, should provide an improved *in vitro* model of dopamine neurons for research on Parkinson’s disease.

## Abbreviations

6-OHDA: 6-hydroxydopamine
ChAT: choline acetyltransferase
DAPI: diamidinophenylindole
DAT: dopamine transporter
DβH: dopamine-β-hydroxylase
En1: engrailed 1
FoxA2: forkhead box A2
H_2_O_2_: hydrogen peroxide
MPP+: 1-methyl-4-phenylpyridinium
MTT: methylthiazoletetrazolium
Nurr1: the nuclear receptor related 1 protein
PD: Parkinson’s disease
Pitx3: paired-like homeodomain 3
TH: tyrosine hydroxylase
Tuj1: β-III tubulin
vGluT1: vesicular glutamate transporter 1
VMAT2: vesicular monoamine transporter 2
